# Association of Sarcopenia and A Body Shape Index With Overall and Cause-Specific Mortality

**DOI:** 10.3389/fendo.2022.839074

**Published:** 2022-07-05

**Authors:** Yu-Shun Qiao, Xingyao Tang, Yin-He Chai, Hong-Jian Gong, Xin Zhang, Coen D. A. Stehouwer, Jian-Bo Zhou

**Affiliations:** ^1^Department of Endocrinology, Beijing Tongren Hospital, Capital Medical University, Beijing, China; ^2^Beijing Tongren Hospital, Capital Medical University, Beijing, China; ^3^Department of Internal Medicine and CARIM School for Cardiovascular Diseases, Maastricht University Medical Center, Maastricht, Netherlands

**Keywords:** sarcopenia, A Body Shape Index, all-cause mortality, cardiovascular mortality, observational study

## Abstract

**Aim:**

This observational study aimed to examine the association between the A Body Shape Index (ABSI) and/or sarcopenia and total, cardiovascular, and cancer mortality.

**Methods:**

The associations of sarcopenia and ABSI with all-cause, cardiovascular, and cancer mortality were assessed in 4,488 participants from the 1999–2004 National Health and Nutrition Examination Survey (NHANES) who were followed up until December 31, 2015. Models were analyzed separately for men and women and adjusted for age, race, and other confounding factors. ABSI was assessed as a continuous measurement by quartile for men and women. Population attributable fractions (PAFs) were calculated to assess mortality caused by sarcopenia and/or ABSI in the study population.

**Results:**

When ABSI was assessed as a continuous variable, the ABSI quartile showed a linear trend for total (*p* = 0.0001), cardiovascular (*p* = 0.04), and cancer (*p* = 0.02) mortality in men and for total (*p* = 0.06) and cardiovascular (*p* = 0.06) mortality in women. The hazard ratios (HRs) of the fourth ABSI quartile were 1.51 [95% confidence interval (CI): 1.20–1.89] in men and 1.23 (95% CI: 0.93–1.64) in women, compared with those in the first quartile. When ABSI was assessed by quartile, the appendicular skeletal mass index (ASMI) was lower in the groups with high ABSI. When high ABSI was combined with sarcopenia, the HRs of all-cause mortality were 2.05 (95% CI: 1.60–2.62) in men and 1.51 (95% CI: 1.19–1.92) in women. In the subpopulation (sarcopenia group or higher ABSI), the PAFs of mortality due to sarcopenia were 26.16% (95% CI: 12.68–37.56) in men and 21.89% (95% CI: 5.64–35.35) in women, and the PAF of mortality due to higher ABSI was 23.70% (95% CI: 12.11–33.77) in men.

**Conclusion:**

The ABSI value was significantly associated with all-cause and cardiovascular mortality, and the co-existence of higher ABSI values and sarcopenia can contribute to a more significant death risk in comparison with high ABSI values or sarcopenia. Moreover, the ABSI values in combination with the ASMI can be used to preliminarily evaluate the content and distribution of fat and muscle and to predict the risk of death in obese and sarcopenic populations.

## Introduction

Obesity is characterized by an abnormal or extensive accumulation of adipose tissue. According to the World Health Organization (WHO) categories, obesity is defined as a body mass index (BMI) [weight/height^3^ (kg/m^2^)] of ≥30 kg/m^2^ (1). The global incidence rate of overweight and obesity has doubled since 1980, and almost one-third of the world’s population is now classified as overweight or obese (2). Obesity has been a heated topic in research as a risk factor for mortality (3), which can cause a series of adverse events, such as cardiovascular disease (CVD), type 2 diabetes, and cancers (4–9), affecting the lifetime and increasing mortality among older people. In recent years, attention has been paid to sarcopenia and the accompanying decline in muscle mass, strength, and performance. Due to regional and age-related variations, the prevalence of sarcopenia ranges from 1% to 29% in the community living population and from 14% to 33% in the long-term care population (10). In the 1999–2004 National Health and Nutrition Examination Survey (NHANES), the prevalence of sarcopenia was approximately 23%–30% in adults ≥60 years old (11). Appendicular skeletal mass (ASM) is commonly applied to reflect skeletal muscle mass, and in the criteria for commonly used definitions of sarcopenia, the appendicular skeletal mass index (ASMI), which is based on ASM after adjusting height, is most widely used (12). Muscle loss is associated with the incidence of death (13, 14), cardiovascular disease (15), and cancer mortality (16).

Based on the aforementioned characteristics of obesity and sarcopenia, sarcopenic obesity (17) has risen as a new consideration. According to previous studies, the average prevalence of sarcopenic obesity ranges from 5% to 10% in older adults (18). Regarding the correlation between sarcopenia and obesity, we can boldly speculate that patients with obesity tend to have limited mobility, and because the body may be too heavy to exercise effectively, muscle mass declines. In turn, the decline of muscle mass weakens metabolic capacity, and the subsequent intake of calories is easily converted into fat accumulation in the body, forming a vicious cycle. Therefore, sarcopenia and obesity can co-occur and are synergistically associated with greater functional decline and higher mortality than either condition alone (19, 20).

With aging, loss of height and muscle mass and increment of fat mass separate the associations between BMI and obesity and weaken the relationship with mortality (21). Therefore, it is important to choose an appropriate anthropometric measure that reflects body composition to better predict mortality. Body composition refers to the amount and distribution of fat and fat-free tissues in the body (1). However, multiple anthropometric measures do not reflect body composition effectively or are easily affected by other body factors, such as BMI, waist circumference (WC), waist-to-height ratio (WtHR), and waist-to-hip ratio (WHR) (22–26). Recently, the A Body Shape Index (ABSI) has been introduced as a new anthropometric measure (27). ABSI is based on WC adjusted for height and weight, which can better explain the degree of central abdominal adiposity (28, 29). Previous studies have shown that ABSI is more strongly associated with mortality than BMI or WC (6, 29–31). However, the joint effect of higher ABSI values and sarcopenia on mortality is unclear. This study was the first to evaluate whether the joint effect of higher ABSI values and sarcopenia led to a higher risk of total, cardiovascular, and cancer mortality compared with participants with higher ABSI values or sarcopenia. The findings from this study can be helpful in assessing the capacity of considering both fat and muscle mass to screen high-risk populations and provide strategies regarding health management and population-level interventions.

## Materials and Methods

### Study Design and Population

This cross-sectional data were acquired from the 1999–2004 NHANES. We then conducted a reanalysis of the data. The NHANES, which represents a noninstitutionalized adult population in the United States, adopted a complex, stratified, multistage probability sampling design that was used to select an appropriate sample of 31,126 participants. Data on mortality were collected from the baseline assessment until December 31, 2015, and the follow-up duration ranged from 11 to 16 years. The mortality data and details were matched between the NHANES and the records of death certificates from the National Death Index. Specific information on the NHANES and mortality data are available online. This study was not subject to review by the local institutional review board because of the deidentified nature of the analyzed data.

The inclusion criteria were as follows (1): the ages of the participants from the 1999–2004 NHANES was ≥60 years (2); there were data on the appendicular skeletal muscle, height, and waist circumference of the included participants; and (3) the participants were followed-up until 2015. Exclusion criteria were as follows: (1) individuals whose weight was >136 kg or whose height was >192 cm since the dual-energy X-ray absorptiometry (DXA) scan had limits on height (maximum, 192.5 cm) and weight (maximum, 136.4 kg); (2) pregnant women and individuals allergic to contrast agents; and (3) individuals who had been exposed to contrast agents or radioactive therapy in the previous 7 days.

### Body Composition Variables

Data on the height (cm), weight (kg), and WC (cm) of the included individuals were directly obtained from the 1999–2004 NHANES. Height (cm) and weight (kg) were measured exactly to one decimal place, and WC was measured midway between the lower rib margin and the iliac crest in a standing condition. These indices were measured by trained researchers to evaluate ABSI. ABSI was developed to reflect abdominal obesity, considering BMI, WC, and height. Based on the height (cm), weight (kg), and WC (cm), ABSI was calculated as follows: ABSI = WC/(BMI^2/3^ × height^1/2^) (27). The ABSI values were assessed as continuous variables and sex-specific quartiles.

ASM was defined as the total muscle mass of both legs and arms, measured using a DXA QDR-4500 Hologic scanner. ASM is estimated, and in most cases, adjustment for height is used to define cutoff values for the ASMI. Based on ASM (kg) and height (m), the ASMI was calculated as ASMI = ASM/height^2^. Referring to the criteria of the European Working Group on Sarcopenia in Older People (EWGSOP), sarcopenia is assessed by the ASMI values (ASM/height^2^ of ≤7.26 kg/m^2^ in men and ≤5.5 kg/m^2^ in women). In addition to false teeth and hearing aids, metal objects were removed. ASM was measured in each participant. Each NHANES cycle consisted of a similar procedure(12).

### Covariates

Demographic characteristics (age, sex, and ethnicity), socioeconomic background (annual household income and educational background), and complications were assessed using self-reported questionnaires. All races were included, such as non-Hispanic White, non-Hispanic Black, Hispanic, and other ethnicities, and we counted the proportion of Black participants separately. In addition, the economic level was reported as annual household income and was ultimately grouped as having achieved an income US$≥65,000. The education level was based on the highest degree and was classified as having an undergraduate degree or above. All comorbidities were based on self-reported questionnaires. Participants with diabetes mellitus were defined as those who had a physician diagnosis while not pregnant, were using insulin or oral hypoglycemic medications, or had a glycohemoglobin level of ≥6.5%. Hypertension was defined as systolic blood pressure of >140 mmHg, diastolic blood pressure of >90 mmHg, or the use of antihypertensive medications. Cardiovascular disease was defined by the self-report of a physician’s diagnosis of hypertension, congestive heart failure, coronary artery disease (CAD), angina, or myocardial infarction.

### Statistical Analysis

All statistical analyses were performed using STATA version 15.0 (Stata Corporation, College Station, TX, USA). Given the known sex differences in death rates, all analyses were performed for men and women.

The risk of death due to high ABSI was assessed using Cox proportional hazards regression models. The size of the risk was reflected by the hazard ratios (HRs). Potential confounders considered in this study were age, race, annual household income, educational background, diabetes, hypertension, CAD, stroke, and the ASMI. We initially adjusted Model 1 for age and race between men and women. Model 2 was adjusted for social background and traditional risk factors, including age, race, annual household income, educational background, diabetes, hypertension, CAD, and stroke. Based on Model 2, Model 3 was obtained by adjusting for the ASMI. Cox regression analyses were performed to examine the independent association between ABSI and all-cause and cause-specific mortality in each model.

As the linear association between ABSI and mortality is not clear, tests for linearity in trends across quartiles were performed for ABSI by fitting Cox models. These studies used the median ABSI per quartile for linear trends. To further verify the predictive power of ABSI for all-cause, cardiovascular, and cancer mortality, the HRs for all-cause, cardiovascular, and cancer mortality were calculated per 1-SD increase in ABSI for each model.

In addition, we calculated the population attributable fractions (PAFs) (%) to indicate the proportion of mortality that could have been prevented in the population in the absence of high ABSI and/or sarcopenia. We used the formula PAF = P_pop_(RR−1) ÷ [(P_pop_ × (RR−1) + 1], where the notation P_pop_ is the proportion of persons in the population exposed to high ABSI and/or sarcopenia, and RR is the relative risk in the exposed group compared with the unexposed group, assessed with the HRs. We calculated the two-sided 95% CIs for the PAFs of ABSI and sarcopenia with mortality. Statistical significance was defined as *p* < 0.05.

## Results

### Baseline Characteristics of Each Study Group

**Figure 1:**
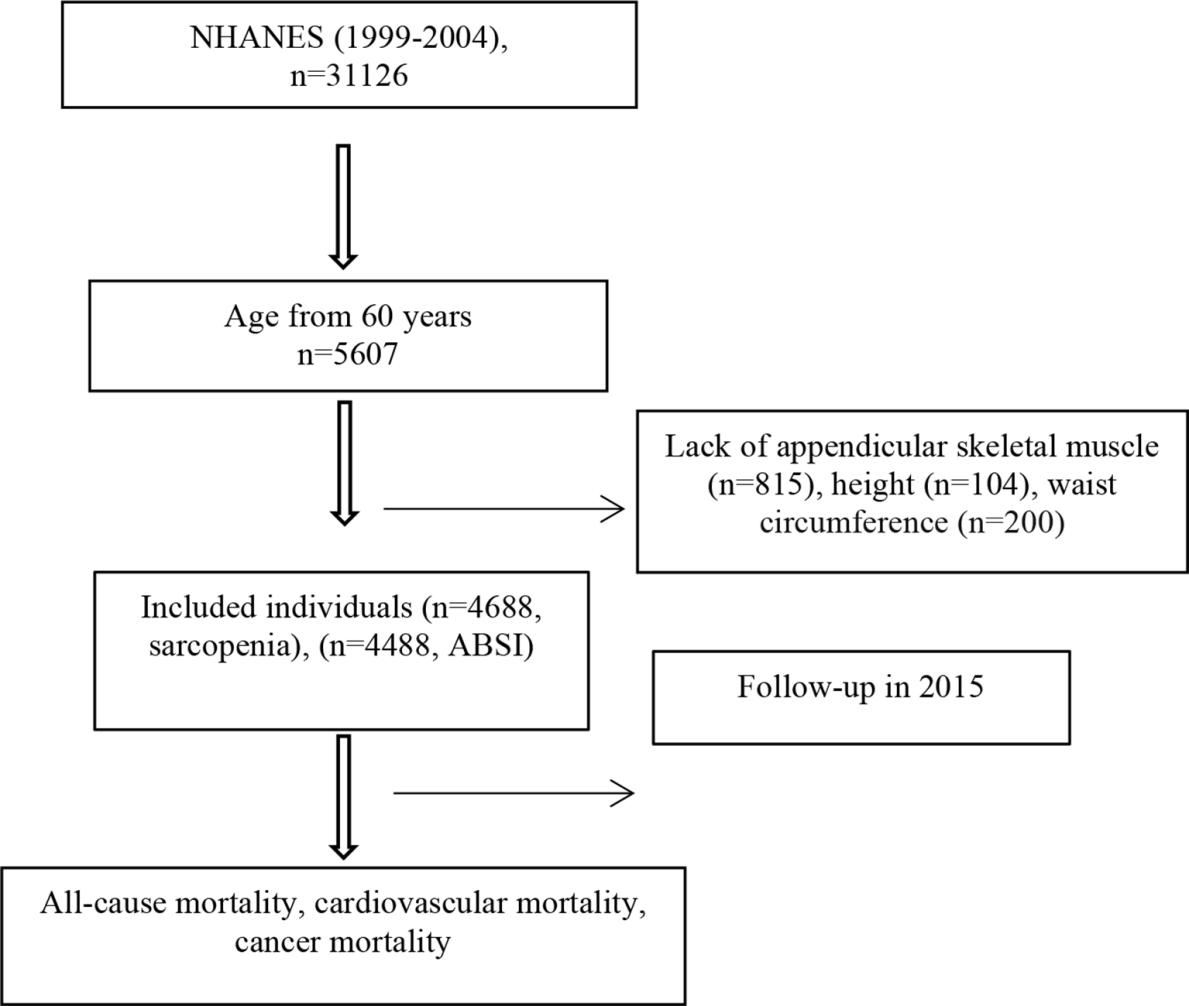
Study chart for analysis (*n* = 4,688).

Of the 31,126 participants screened, we limited the sample to individuals aged ≥60 years (*n* = 5,607), excluding 815 participants without records of appendicular skeletal muscle mass, 104 participants without records of height, and 200 participants without records of WC. Ultimately, 4,488 participants were enrolled (**Figure 1**). The ABSI values were assessed as continuous variables and quartiles. The HRs for all-cause, cardiovascular, and cancer mortality were calculated using the first quartile as a reference. **Table 1** shows the characteristics of each group. Among men and women, age was positively associated with the ABSI values. In contrast, ASMI and the proportion of Black individuals were negatively associated with the ABSI values, regardless of sex. In women, the prevalence of diabetes, hypertension, and CAD was related to increased ABSI; however, these relationships were not observed in men. Compared with women, men had a significantly higher annual household income, educational background, ASMI values, and prevalence of diabetes and CAD, whereas women were slightly older and had a higher frequency of hypertension.

**Table 1:** Associations between ABSI and risk of all-cause mortality and cause-specific mortality among individuals aged ≥60 years (*n* = 4,488).

ABSI quartile	Q1 (reference) (*n* = 554 men, *n* = 569 women)	Q2 (*n* = 553 men, *n* = 568 women)	Q3 (*n* = 554 men, *n* = 569 women)	Q4 (*n* = 553 men, *n* = 568 female)	*p*-value
**Men (*n* = 2,214)**					
Age [mean (SE), year]	67.90 (0.4)	69.10 (0.4)	70.30 (0.3)	73.10 (0.3)	<0.000
Black [*n* (%)]	78 (14.15)	39 (7.03)	30 (5.50)	17 (3.16)	<0.000
Annual household income ≥$65,000 [*n* (%)]	184 (33.21)	133 (24.12)	100 (18.05)	89 (16.08)	<0.000
Education—college graduate or above [*n* (%)]	116 (30.00)	131 (23.67)	147 (26.50)	128 (23.13)	0.001
Diabetes [*n* (%)]	121 (21.90)	118 (21.30)	138 (24.90)	135 (24.40)	0.22
Hypertension [*n* (%)]	334 (60.30)	322 (58.30)	372 (67.20)	369 (66.70)	0.05
Stroke [*n* (%)]	40 (7.30)	29 (5.20)	33 (6.00)	40 (7.30)	0.61
CAD (*n* (%)]	79 (14.30)	91 (16.40)	95 (17.20)	71 (12.80)	0.57
ASMI [mean (SE)]	8.49 (0.06)	8.15 (0.07)	7.93 (0.07)	7.40 (0.05)	<0.000
ABSI [mean (SE)]	0.081 (0.00)	0.085 (0.00)	0.087 (0.00)	0.090 (0.00)	<0.000
**Women (*n* = 2,274)**					
Age [mean (SE), year]	68.90 (0.4)	70.90 (0.3)	71.00 (0.4)	73.60 (0.3)	<0.000
Black [*n* (%)]	73 (12.80)	47 (8.20)	39 (6.90)	30 (5.30)	<0.000
Annual household income ≥$65000 [*n* (%)]	91 (16.00)	106 (18.60)	77 (13.60)	64 (11.20)	<0.000
Education—college graduate or above [*n* (%)]	75 (13.20)	93 (16.40)	71 (12.50)	62 (10.90)	0.001
Diabetes [*n* (%)]	59 (10.40)	84 (14.80)	116 (20.40)	165 (29.10)	<0.000
Hypertension [*n* (%)]	380 (66.80)	390 (68.60)	440 (77.30)	447 (78.70)	<0.000
Stroke [*n* (%)]	32 (5.60)	29 (5.10)	34 (6.00)	48 (8.50)	0.10
CAD [*n* (%)]	31 (5.50)	43 (7.50)	46 (8.10)	51 (9.00)	0.02
ASMI [mean (SE)]	6.65 (0.07)	6.46 (0.04)	6.36 (0.05)	6.05 (0.06)	<0.000
ABSI mean (SE)	0.076 (0.00)	0.082 (0.00)	0.085 (0.00)	0.090 (0.00)	<0.000

### Association Between ABSI and Mortality

**Table 2** shows the HRs and 95% CIs for quartiles of ABSI and trend tests for linearity after adjustment for age and race (Model 1), after additional adjustment for confounders (Model 2), and after adjustment for Model 2 and the ASMI (Model 3).

**Table 2:** Association of ABSI with all-cause and cause-specific mortality.

Quartile for body fat	Q1 (reference)	Q2	Q3	Q4	*p*-value	Each SD increment
**Men**						
All-cause mortality						
Model 1 [HR (95% CI)]	Reference	1.02 (0.83–1.26)	1.48 (1.17–1.88)	1.84 (1.44–2.34)	0.0000	1.18e+31 (1.04e+22; 1.34e+40; *p* < 0.000)
Model 2 [HR (95% CI)]	Reference	0.99 (0.76–1.30)	1.44 (1.13–1.86)	1.68 (1.31–2.16)	0.0000	6.69e+27 (9.15e+18; 4.90e+36; *p* < 0.000)
Model 3 [HR (95% CI)]	Reference	0.96 (0.74–1.26)	1.36 (1.06–1.76)	1.51 (1.20–1.89)	0.0001	4.93e+22 (2.92e+14; 8.33e+30; *p* < 0.000)
Cardiovascular mortality						
Model 1 [HR (95% CI)]	Reference	0.82 (0.48–1.38)	1.38 (0.87–2.19)	1.75 (1.10–2.77)	0.0033	1.29e+40 (1.31e+21; 1.27e+59; *p* < 0.000)
Model 2 [HR (95% CI)]	Reference	0.75 (0.44–1.29)	1.28 (0.82–2.00)	1.66 (1.06–2.60)	0.0071	4.29e+38 (8.98e+162.05e+60; *p* = 0.001)
Model 3 [HR (95% CI)]	Reference	0.71 (0.42–1.20)	1.14 (0.75–1.74)	1.37 (0.91–2.07)	0.043	3.24e+29 (2.78e+09; 3.78e+49; *p* = 0.005)
Cancer mortality						
Model 1 [HR (95% CI)]	Reference	1.02 (0.69–1.52)	1.496 (0.95–2.35)	1.97 (1.32–2.96)	0.0004	5.42e+40 (1.15e+23; 2.54e+58; *p* < 0.000)
Model 2 [HR (95% CI)]	Reference	1.02 (0.64–1.64)	1.55 (0.91–2.65)	1.97 (1.21–3.20)	0.0023	7.22e+42 (8.11e+22; 6.42e+62; *p* < 0.000)
Model 3 [HR (95% CI)]	Reference	0.97 (0.61–1.55)	1.41 (0.82–2.43)	1.63 (0.99–2.68)	0.0239	1.77e+34 (1.06e+13; 2.96e+55; *p* = 0.002)
**Women**						
All-cause mortality						
Model 1 [HR (95% CI)]	Reference	0.97 (0.76–1.24)	1.15 (0.93–1.40)	1.38 (1.10–1.74)	0.0009	1.79e+12 (1,682,233; 1.90e+18; *p* < 0.000)
Model 2 [HR (95% CI)]	Reference	0.92 (0.71–1.20)	1.09 (0.86–1.39)	1.26 (0.95–1.67)	0.046	4.42e+09 (49.53483; 3.95e+17; *p* = 0.02)
Model 3 [HR (95% CI)]	Reference	0.92 (0.71–1.19)	1.08 (0.85–1.38)	1.23 (0.93–1.64)	0.065	8.85e+08 (6.791029; 1.15e+17; *p* = 0.03)
Cardiovascular mortality						
Model 1 [HR (95% CI)]	Reference	1.209 (0.64–2.30)	1.61 (0.86–3.02)	2.11 (1.24–3.58)	0.0034	9.90e+25 (5.16e+12; 1.90e+39; *p* < 0.000)
Model 2 [HR (95% CI)]	Reference	1.09 (0.49–2.41)	1.57 (0.73–3.38)	1.75 (0.86–3.57)	0.070	3.95e+21 (5,314.441; 2.93e+39; *p* = 0.02)
Model 3 [HR (95% CI)]	Reference	1.10 (0.50–2.42)	1.60 (0.74–3.45)	1.81 (0.88–3.71)	0.064	1.45e+22 (554.1214; 3.80e+41; *p* = 0.03)
Cancer mortality						
Model 1 [HR (95% CI)]	Reference	0.61 (0.37–0.99)	1.16 (0.69–1.95)	1.37 (0.74–2.54)	0.107	1.30e+15 (0.0001678; 1.00e+34; *p* = 0.11)
Model 2 [HR (95% CI)]	Reference	0.59 (0.35–1.01)	0.93 (0.54–1.61)	1.14 (0.59–2.18)	0.418	6.70e+09 (1.70e−10; 2.63e+29; *p* = 0.32
Model 3 [HR (95% CI)]	Reference	0.60 (0.34–1.00)	1.02 (0.59–1.76)	1.24 (0.67–2.30)	0.22	1.07e+11 (1.70e−08; 6.74e+29; *p* = 0.24

Model 1: adjusted for age and race/ethnicity.

Model 2: additionally adjusted for annual household income, education, diabetes, hypertension, stroke, and coronary artery disease.

Model 3: Model 2 plus ASMI.

In models assessing ABSI, there were significant linear associations for men, regardless of the model (Models 1, 2, and 3). In each model, the fourth quartile showed the highest HR. The associations between ABSI and all-cause, cardiovascular, and cancer mortality risk were significant in men after full adjustment (Model 3), and the HRs with 95% CI for Models 1, 2, and 3 were 1.51 (1.20–1.89), 1.37 (0.91–2.07), and 1.63 (0.99–2.68), respectively. However, the linear associations in women were not as significant as those in men; linear associations, which were significant or close to statistical significance, were only shown in Models 1, 2, and 3 of the all-cause and cardiovascular mortality groups. There was no association between ABSI and cancer mortality among the women.

### Joint Association of ABSI and Sarcopenia With Mortality

**Table 3** shows that, compared with the control group (nonsarcopenia and low ABSI), both sarcopenia and high ABSI independently increased the risk of all-cause, cardiovascular, and cancer mortality in men, based on Model 2. When participants with high ABSI suffered from sarcopenia, the HRs of each mortality event rose more sharply. These associations were also exhibited in the female study group involving all-cause mortality (HR = 1.51, 95% CI: 1.19–1.92) rather than cardiovascular (HR = 1.85, 95% CI: 1.07–3.19) and cancer (HR = 1.37, 95% CI: 0.70–2.66) mortality. In addition, **Supplementary Table S1** shows the addictive interaction between ABSI and sarcopenia on mortality, and the relative excess risk of interaction (RERI) was calculated but lacked statistical significance, regardless of sex.

**Table 3:** Joint association of ABSI and sarcopenia (SA) with all-cause and cause-specific mortality.

	Non-Sa, Low ABSI (0)	Non-SA, High ABSI (1)	SA, Low ABSI (2)	SA, High ABSI (3)
**Men**				
All-cause mortality				
Model 2 [HR (95% CI)]	Reference	1.66 (1.35–2.03)	1.81 (1.38–2.38)	2.05 (1.60–2.62)
Cardiovascular mortality				
Model 2 [HR (95% CI)]	Reference	1.44 (0.90–2.29)	1.52 (0.90–2.58)	2.49 (1.61–3.87)
Cancer mortality				
Model 2 [HR (95% CI)]	Reference	1.70 (1.05–2.73)	2.37 (1.34–4.19)	3.01 (1.87–4.82)
**Women**				
All-cause mortality				
Model 2 [HR (95% CI)]	Reference	1.28 (1.07–1.53)	1.48 (1.07–2.03)	1.51 (1.19–1.92)
Cardiovascular mortality				
Model 2 [HR (95% CI)]	Reference	1.65 (1.02–2.67)	1.49 (0.48–4.69)	1.85 (1.07–3.19)
Cancer mortality				
Model 2 [HR (95% CI)]	Reference	1.32 (0.89–1.97)	0.55 (0.20–1.51)	1.37 (0.70–2.66)

Model 1 was adjusted for age and race/ethnicity, while Model 2 was additionally adjusted for annual household income, education, diabetes, hypertension, stroke, and coronary artery disease.

Sarcopenia and ABSI were split at their respective median values.

### Sex-Specific PAFs of Sarcopenia to Mortality

**Table 4** shows that 8.225% (95% CI: 4.247–12.04) of male deaths can be attributed to sarcopenia in the included total male population, and the proportion reached 26.16% (95% CI: 12.68–37.56) among the male sarcopenia group. This ratio was respectively 6.88% (2.21–11.41) and 21.89% (5.64–35.35) in the included total female participants and female participants with sarcopenia. In addition, the impact of sarcopenia on cardiovascular and cancer mortality may be sex specific. **Table 4** indicates that the presence of sarcopenia in men is the leading cause of cardiovascular and cancer mortality, regardless of the total or subpopulation, but this association was not statistically significant in women.

**Table 4:** Sex-specific PAFs of sarcopenia to all-cause and cause-specific mortality in the US population.

	PAFa (%) 95% CI: total population	PAFa (%) 95% CI: subpopulation^a^	PAFa (%) 95% CI: total population	PAFa (%) 95% CI: subpopulation^a^
	Men		Women	
All causes	8.23 (4.25–12.04); *p* < 0.000	26.16 (12.68–37.56); *p* < 0.000	6.88 (2.12–11.41); *p* = 0.006	21.89 (5.64–35.35); *p* = 0.01
Cardiovascular disease	12.75 (5.31–19.60); *p* = 0.002	37.57 (12.57–55.42); *p* = 0.007	3.35 (−9.00–14.31); *p* = 0.57	12.93 (−45.67–47.95); *p* = 0.59
Cancer	18.90 (11.29–25.87); *p* < 0.000	46.06 (25.06–61.17); *p* < 0.000	−3.49 (−14.92–6.80); *p* = 0.51	21.54 (−110.58–29.85); *p* = 0.48

^a^Adjusted for age, ethnicity, annual household income, education, diabetes, hypertension, stroke, coronary artery disease, and ABSI.

^a^Scenario 1: Subpopulation representing the sarcopenia group.

### Sex-Specific PAFs of ABSI to Mortality

**Table 5** shows that when high ABSI was eliminated as the risk factor, all-cause and cancer mortality respectively decreased by 13.23% (6.99–19.05) and 18.27% (3.31–30.92) among the total male population. However, in the male subpopulation (lower ABSI group), this degree of risk reduction for all-cause and cancer mortality reached 23.70% (12.11–33.77) and 31.74% (3.17–51.88), respectively. In women, high ABSI was not an attributable factor for all-cause (PAF = 10.91%, 95% CI: −3.72–23.48, *p* = 0.13), cardiovascular (PAF = 29.62%, 95% CI: −5.69–53.14, *p* = 0.09), and cancer (PAF = 33.49%, 95% CI: −1.04–56.22, *p* = 0.06) mortality.

**Table 5:** Sex-specific PAFs of ABSI to all-cause and cause-specific mortality in the US population.

	PAFa (%) 95% CI: total population	PAFa (%) 95% CI: subpopulation^a^	PAFa (%) 95% CI: total population	PAFa (%) 95% CI: subpopulation^a^
	Men		Women	
All causes	13.23 (6.99–19.05); *p* < 0.000	23.70 (12.11–33.77); *p* < 0.000	6.09 (−1.78–13.36); *p* = 0.12	10.91 (−3.72–23.48); *p* = 0.13
Cardiovascular disease	13.53 (−2.03–26.72); *p* = 0.08	24.38 (−6.35–46.23); *p* = 0.11	17.53 (−1.27–32.84); *p* = 0.06	29.62 (−5.69–53.14); *p* = 0.09
Cancer	18.27 (3.31–30.92); *p* = 0.02	31.74 (3.17–51.88); *p* = 0.03	18.54 (1.59–32.57); *p* = 0.03	33.49 (−1.04–56.22); *p* = 0.06

^a^Adjusted for age, ethnicity, annual household income, education, diabetes, hypertension, stroke, and coronary artery disease.

^a^Scenario 1: Subpopulation represents the lower ABSI group.

## Discussion

Our main finding is that after adjusting for multiple factors, with increasing ABSI, total and cause-specific mortality of male participants as well as total and cardiovascular mortality of female participants significantly increased. More importantly, when higher ABSI was integrated with sarcopenia, the mortality rates in men were significantly higher than those caused by a single factor (higher ABSI *or* sarcopenia). However, only all-cause mortality was consistent with this association among women.

Previous studies (4, 32–35) indicated racial differences in the predictive mortality power of ABSI, which was originally formulated based on data from White, Black, and Hispanic individuals (27). Our data suggested that as ABSI increased, the proportion of Black participants gradually decreased from the first to the fourth quartile, which indicated that the body composition of Black participants had a generally lower ABSI. Previous studies have shown that older Black men and women generally possess higher appendicular lean mass and BMI than older White men and women (36–38). Our data also covered the educational background and annual household income of participants; therefore, social and economic factors can also influence the proportion of Black individuals among groups (38), which is worthy of further study.

The impact of ABSI on mortality may be sex-specific in this study, similar to previous reports (35, 39, 40). Specifically, we found that the relationship between ABSI and all-cause and cause-specific mortality was not significant in women. Some speculation and facts could explain why ABSI may not be an appropriate predictor of the risk of death in women. First, the men in our study had higher baseline ABSI levels and a higher prevalence of diabetes mellitus. Second, there was a lower frequency of cardiovascular events in women (Q1, 5.5%; Q2, 7.5%; Q3, 8.1%; Q4, 9.0%) than in men (Q1, 14.3%; Q2, 16.4%; Q3, 17.2%; Q4, 12.8%), and the subsequent statistical power might be lower, which could explain the lack of the associations among women. Third, the span of the ABSI values in female participants was larger than that in male participants, and ABSI was highly clustered around the mean with relatively small variance (41). Therefore, when the span is large and the number of groups is small, some potential connections may be obscured. Finally, the ABSI was originally designed to assess the impact of central obesity (6), since the location of fat accumulation is more important than the actual amount of body fat (14). Due to differences in fat distribution between men and women, more central fat is deposited among men with age (42). This could be one reason why the association between ABSI and mortality was not observed among women.

There are some possible explanations for ABSI’s predictive power for total and cause-specific mortality. First, the distribution of body fat is more predictable than overall obesity in predicting death (43), and ABSI can better reflect central obesity than WC, BMI, or WHR (6). Second, age is a crucial factor in the assessment of the death rate, and aging is frequently accompanied by an increase in visceral fat and a decrease in muscle mass (44). ABSI tends to increase significantly with aging (27). In summary, ABSI better reflects adenoidal obesity and age, and therefore, it better predicts mortality.

In this study, AMSI gradually decreased from the first to the fourth quartile of ABSI, regardless of sex, which indicated that muscle loss tended to accompany the accumulation of adipose tissue. When both coexist, they can synergistically increase the risk of death (19). This is consistent with the results in **Table 4**. There are several common mechanisms underlying sarcopenia and obesity. First, the increased generation of diverse substances from fat tissue, such as tumor necrosis factor-α (TNF-α) and leptin, can affect insulin resistance and energy metabolism, leading to the loss of muscle mass and the deposition of body fat (45). Second, age was a significant factor. All participants were aged ≥60 years, as significant age-related differences exist in the body compositions of men and women aged ≥60 years, such as loss of muscle mass and an increase in fat mass (46–48). Third, C-reactive protein (CRP) and interleukin-6 (IL-6), which have been demonstrated to be important predictors of muscle loss in people aged ≥55 years old, have been shown to be positively associated with fat mass and negatively associated with muscle mass (49, 50). Muscle loss and fat increase, including but not limited to the above mechanisms, produce a vicious cycle, which further leads to an increased risk of all-cause, cardiovascular, and cancer mortality (6, 51, 52).

The strength of the present study was that the joint association of ABSI and sarcopenia with all-cause and cause-specific mortality was evaluated for the first time, and this joint association may be sex-specific. The outcome of our study is that individuals who suffer from sarcopenia and high ABSI have a significantly higher risk of death than those without sarcopenia or with low ABSI, which stresses the urgency for a scientifically rational assessment of obesity and sarcopenia. ABSI can better assess abdominal obesity, and ASMI can evaluate lean mass. The combination of both can significantly increase the predictive power of mortality, thereby facilitating more targeted interventions to improve quality of life and extend life expectancy among older people. Current evidence suggests that progressive resistance exercise (RE) and aerobic exercise (AE) effectively reduce the risk of obesity and sarcopenia (53–55). Consequently, comprehensive assessment, diagnosis, and treatment of obesity and sarcopenia should be more greatly considered for hospitalized patients to reduce the incidence of adverse events and improve their prognosis.

Our study had some limitations. First, certain essential information regarding preexisting disease interventions, such as antihypertensive drugs, antidiabetic drugs, and CAD treatment, is unavailable and is often associated with study outcomes. Second, the sample size of women was not large enough to ensure that the study results lacked statistical significance. Third, smoking, alcohol consumption, physical activity, and dietary quality were not considered important confounding factors. Fourth, there were no other anthropometric measures of the participants for comparison, such as BMI and WC.

In conclusion, the joint effect of high ABSI and sarcopenia is strongly associated with all-cause and cause-specific mortality, compared with the impact of high ABSI or sarcopenia alone. ABSI and ASMI may be appropriate indices for assessing the proportionate relationship between adiposity with sarcopenia and mortality and should be considered for use in routine clinical care and as intervention indices as a result. However, the use of these indicators to assess female mortality requires further investigation and discussion.

## Data Availability Statement

The original contributions presented in the study are included in the article/**Supplementary Material**. Further inquiries can be directed to the corresponding authors.

## Ethics Statement

This article does not contain any studies with human participants or animals performed by any of the authors.

## Author Contributions

The authors are solely responsible for the design and conduct of this study, all the study analyses, the drafting and editing of the manuscript, and its final contents. J-BZ is the guarantor of this work and, as such, had full access to all the data in the study and takes responsibility for the integrity of the data and the accuracy of the data analysis. All authors listed have made a substantial, direct, and intellectual contribution to the work and approved it for publication.

## Funding

This work was supported by the National Natural Science Foundation of China (No. 82070851, 81870556), Beijing Municipal Administration of Hospital’s Youth Program (QML20170204), and Excellent Talents in the Dongcheng District of Beijing.

## Conflict of Interest

The authors declare that the research was conducted in the absence of any commercial or financial relationships that could be construed as a potential conflict of interest.

## Publisher’s Note

All claims expressed in this article are solely those of the authors and do not necessarily represent those of their affiliated organizations, or those of the publisher, the editors and the reviewers. Any product that may be evaluated in this article, or claim that may be made by its manufacturer, is not guaranteed or endorsed by the publisher.

## Supplementary Material

Supplementary Table 1 Attributing effects of the additive interaction between sarcopenia and ABSI on the risks of all-cause and specific cause mortality. *RERI, Relative excess risk due to interaction. *Multivariable adjusted hazard ratios estimated from Cox models. ‡Adjusted for age, ethnicity, annual household income, education, diabetes, hypertension, stroke, and coronary artery disease.

## Abbreviations

ABSI, A Body Shape Index; PAF, population attributable fraction; NHANES, National Health and Nutrition Examination Survey; ASMI, appendicular skeletal mass index; BMI, body mass index; WC, waist circumference; WtHR, waist-to-height ratio; WHR, waist-to-hip ratio.
