# Supplementary material for: Association of Sarcopenia and A Body Shape Index With Overall and Cause-Specific Mortality
Source: Front Endocrinol (Lausanne). 2022 Jul 5;13:839074. doi: 10.3389/fendo.2022.839074 (PMC9294172; doi:10.3389/fendo.2022.839074)
Supplement: Supplementary Table 1 — Attributing effects of the additive interaction between sarcopenia and ABSI on the risks of all-cause and specific cause mortality. *RERI, Relative excess risk due to interaction. *Multivariable adjusted hazard ratios estimated from Cox models. ‡Adjusted for age, ethnicity, annual household income, education, diabetes, hypertension, stroke, and coronary artery disease. [file Table_1.docx]

Supplement information

eTable 1. Attributing effects to additive interaction between sarcopenia and ABSI on risks of All-Cause and Cause-Specific Mortality *

eTable 1. Attributing effects to additive interaction between sarcopenia and ABSI on risks of All-Cause and Cause-Specific Mortality *

| RERI | Male | | | Female | | |
| --- | --- | --- | --- | --- | --- | --- |
|  | All-cause‡ | Cardiovascular disease ‡ | Cancer‡ | All-cause‡ | Cardiovascular disease ‡ | Cancer‡ |
| Sarcopenia0-ABSI0 | reference | reference | reference | reference | reference | reference |
| Sarcopenia1-ABSI1 | -0.42(-1.01-0.17) | 0.53(-0.60-1.66) | -0.06(-1.46-1.33) | -0.25(-0.80-0.30) | -0.29(-2.35-1.76) | 0.49(-0.35-1.34) |
| P value | 0.16 | 0.36 | 0.93 | 0.38 | 0.78 | 0.25 |

RERI: Relative excess risk due to interaction

*Multivariable adjusted hazard ratios estimated from Cox models;

‡Adjusted by age, ethnicity, annual household income, education, diabetes, hypertension, stroke, coronary artery diseases.
